# Evaluation of Risk Factors for Revision Surgery After Proximal Femoral Nailing for Intertrochanteric Fractures

**DOI:** 10.3390/medicina61122085

**Published:** 2025-11-22

**Authors:** Evrim Duman, Ömer Torun, Ahmet Berkay Girgin, Mehmet Alperen Özçelik, Ahmet Acar, Hüseyin Bilgehan Çevik

**Affiliations:** 1 Department of Orthopaedics and Traumatology, Ankara Etlik City Hospital, Ankara 06170, Türkiye; evrimduman@gmail.com (E.D.); omertor46@gmail.com (Ö.T.); acar.ahmet.91@gmail.com (A.A.); bilgehancevik@gmail.com (H.B.Ç.); 2 Department of Communicable Diseases and Early Warning, Republic of Türkiye Ministry of Health General Directorate and Public Health, Ankara 06100, Türkiye; alperenozcelik@yahoo.com

**Keywords:** intertrochanteric fracture, proximal femoral nail, revision surgery, implant failure, hip fracture

## Abstract

*Background and Objectives:* Intertrochanteric femur fractures are very common, especially in the elderly population, and cause serious morbidity and mortality. Today, the most commonly used implants in the treatment of these fractures are proximal femoral nails (PFNs). This study aimed to analyze the clinical and radiological results of patients who underwent surgical treatment with a proximal femoral nail (PFN) for intertrochanteric femur fractures and later required revision surgery for various reasons. *Materials and Methods:* Patients who underwent surgical treatment PFN due to intertrochanteric femur fractures between 2022 and 2025 were included in the study, and the patients were divided into revision and non-revision groups. Demographic information, postoperative radiological measurements, complications, and reasons for revision surgery were noted, and risk factors leading to revision were determined using bivariate and multivariate analyses. *Results:* A total of 207 patients, 97 revision (46.9%) and 110 non-revision (53.1%), were included in this study. Cut-out was identified as the most common revision cause (*n* = 52, 53.6%), followed by loss of reduction (*n* = 15, 15.5%), implant failure (*n* = 14, 14.4%), nonunion (*n* = 6, 6.2%), infection (*n* = 4, 4.1%), cut-through (*n* = 3, 3.1%), and avascular necrosis of the femoral head (*n* = 3, 3.1%). When bivariate analysis was performed to identify risk factors for revision, it was observed that female gender (*p* = 0.004), presence of posteromedial comminution (*p* < 0.001), operation under spinal anesthesia (*p* = 0.023), surgery in supine position (*p* < 0.001), using closed reduction techniques (*p* < 0.001), presence of infection (*p* = 0.004), and higher Charlson comorbidity index values (*p* < 0.001) increased the risk of revision. Additionally, positive and neutral medial cortex support (*p* < 0.001) decreased the risk of revision. Multivariate analysis was also applied to the parameters found to be significant in bivariate analysis. As a result of this analysis, surgery in the supine position (*p* < 0.001), using closed reduction techniques (*p* < 0.001), and higher Charlson comorbidity index values (*p* < 0.001) remained significant. *Conclusions:* Careful evaluation of the fracture morphology, ensuring optimal reduction, and considering the accompanying comorbidities of the patients in the surgical planning of unstable trochanteric fractures stand out as key elements in increasing surgical success.

## 1. Introduction

Intertrochanteric femur fractures are injuries that are frequently seen in the elderly population as a result of low-energy trauma due to falls and cause significant morbidity and mortality [1]. These fractures, together with the effects of osteoporosis, stand out as a serious public health problem [2]. Today, surgery is considered the primary treatment for these fractures, and the most commonly preferred implants for fracture fixation include proximal femoral nail (PFN) and sliding hip screw (SHS) [3,4].

However, after surgical treatment, some patients may develop various problems due to mechanical (cut-out, cut-through, implant failure, malposition) or biological (nonunion, avascular necrosis) reasons. Such complications lead to difficult clinical conditions that require revision surgery and negatively affect the quality of life of patients [5,6]. Revision surgery is a procedure that requires high levels of attention in terms of both surgical techniques and postoperative follow-up, and its results depend on various factors [7].

This study aimed to analyze the clinical and radiological results of patients who underwent surgical treatment for intertrochanteric femur fractures and later required revision surgery for various reasons. This analysis aims to determine the main factors that lead to the need for revision, to evaluate the effectiveness of the surgical methods used, and to optimize treatment approaches.

## 2. Materials and Methods

This retrospective study was conducted at a tertiary care center and was approved by the Institutional Review Board in compliance with the Declaration of Helsinki.

### 2.1. Patient Selection

The study included patients who underwent PFN for intertrochanteric femur fractures, defined according to the AO/OTA classification; had at least 6 months of follow-up; and had adequate clinical and radiological data. Patients with pathological fractures, concomitant extremity fractures, or those who did not give consent to participate were excluded. At least 6-months of follow-up was confirmed for each patient according to their last outpatient clinic visit. After retrospective analysis, 277 patients who underwent PFN for intertrochanteric femur fractures between 2022 and 2025 were identified. Of these, 26 patients were excluded from the study due to pathological fracture, 25 patients due to concomitant extremity fracture, and 19 patients because they did not consent to participate. As a result, 207 patients were analyzed. The patients were divided into two groups according to the need for revision surgery: revision (Group 1, *n* = 97) and non-revision (Group 2, *n* = 110). Patients requiring revision surgery were those who were reoperated due to implant failure, nonunion, malposition, implant migration (cut-out, cut-through), or other mechanical complications.

### 2.2. Data Collection and Outcome Assessment

All surgeries were performed at the same center by five different surgical teams with the same level of experience. Surgery in the supine or lateral position was determined according to the preference of the surgical team. All surgeries began with a closed reduction attempt, but when closed reduction was unsuccessful, open reduction was performed. Intraoperative imaging guidance was standardized. Additionally, the type of nail used was determined according to the preference of the surgical team. Parameters such as age, gender, comorbidities, medications of patients, fracture type (AO/OTA classification), operation duration, implant type and size, reduction quality (tip-apex distance-TAD, calcar-referenced tip-apex distance-Cal TAD, Femur cortical thickness index, lateral cortical thickness, ratio of nail length to femur length, lag screw position according to Cleveland–Bosworth quadrants [8], presence of posteromedial comminution, trochanter minor fracture size), revision surgery time and complications, and type of revision were obtained from hospital registry system and the hospital’s Picture Archiving and Communication Systems (PACS). Charlson comorbidity index [9] was calculated according to the collected data of the patients.

### 2.3. Statistical Analysis

A priori power analysis was performed using G*Power 3.1 to determine the required sample size for comparing two independent means. Based on an expected effect size (Cohen’s d) of 0.6, a two-tailed α of 0.05, and a desired power (1 − β) of 0.80, the minimum required sample size was calculated as 98 participants per group.

The research data was evaluated using the Statistical Package for the Social Sciences (SPSS) version 23, IBM Corp., Armonk, NY, USA, statistical package program. In the descriptive statistics section, categorical variables were presented in tables and graphics by giving numbers and percentages, and continuous variables were presented with mean ± standard deviation and median (IQR). The conformity of continuous variables to normal distribution was evaluated using visual (histogram and probability graphs) and analytical methods (Kolmogorov–Smirnov/Shapiro–Wilk tests). The independent samples *t*-test was used in comparisons of continuous variables that conformed to normal distribution between two groups, and the Mann–Whitney U test was used in comparisons of continuous variables that did not conform to normal distribution between two groups. The chi-square test was used in comparisons of categorical variables. Statistical significance level was accepted as *p* < 0.05. Potential collinearity between explanatory variables was assessed prior to regression analyses. Variance inflation factors (VIFs) and correlation matrices were examined to identify overlapping effects. When variables demonstrated high collinearity (VIF > 5 or strong pairwise correlation), only the clinically more relevant variable was retained in the multivariate model to avoid instability of estimates. The dependent variable in our logistic regression analysis was revision status. The independent (explanatory) variables included gender, presence of posteromedial comminution, anesthesia type, patient’s position, reduction, surgical site infection, positive medial cortical support, calcar-referenced tip-apex distance, length of hospital stay, and Charlson comorbidity index. Categorical variables were coded as no/yes, while continuous variables were kept in their original form. Variables with a *p*-value less than 0.25 in the bivariate comparisons were included in the bivariate regression analysis. Variables that were significant in the bivariate regression analysis were then entered into the multivariate logistic regression model. We used the “backward” selection method to identify the most significant variables in the final model.

## 3. Results

A total of 1076 patients who were operated on for interthrocanteric femur fractures were identified between 2022 and 2025, and 207 patients who met the inclusion criteria were included in the study. Of these, 137 patients (66.2%) were female and 70 (33.8%) were male. The median age of the patients was 81 years (IQR, 74–87 years). There were 97 patients (46.9%) in the revision group and 110 patients (53.1%) in the non-revision group.

**Figure 1:**
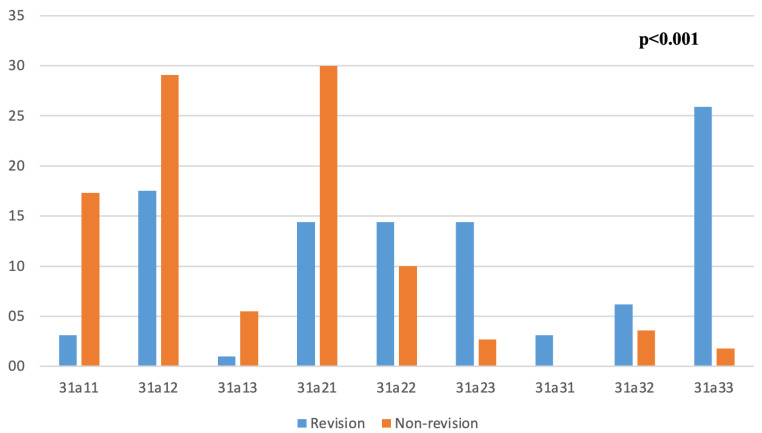
Fracture types according to AO classification and comparison between two groups.

The most common fracture type according to the AO classification was 31a12 (*n* = 49, 23.7%). Fracture types according to AO classification and comparison between two groups are given in detail in Figure 1. The femurs of 110 patients (53.1%) were found to be type C according to the Dorr classification. Posteromedial comminution was seen in 122 of the fractures (58.9%). Lesser trochanter involvement was present in 131 of the fractures (63.3%), and the median fracture size was measured as 4 cm (IQR, 3–5.4 cm). The median lateral cortical thickness was measured as 22 mm (IQR, 14–29 mm). While PFNA was used in 198 fractures (95.7%), long PFNA was used in 9 fractures (4.3%).

A total of 165 patients (79.7%) were operated on under spinal anesthesia, and 42 patients (20.3%) were operated on under general anesthesia. While 130 patients (62.8%) were operated on in the lateral decubitus position, 77 patients (37.2%) were operated on in the supine position. Closed reduction methods were used in 170 patients (82.1%), and the fractures of 37 patients (17.9%) were fixed after open reduction. When these three parameters were compared between the two groups, the results were statistically significant (*p* = 0.021, *p* < 0.001, *p* < 0.001).

The mean Charlson comorbidity index value was calculated as 6.3 ± 1.5 in the revision group and 5.2 ± 1.7 in the non-revision group, and this difference was statistically significant (*p* < 0.001).

**Figure 2:**
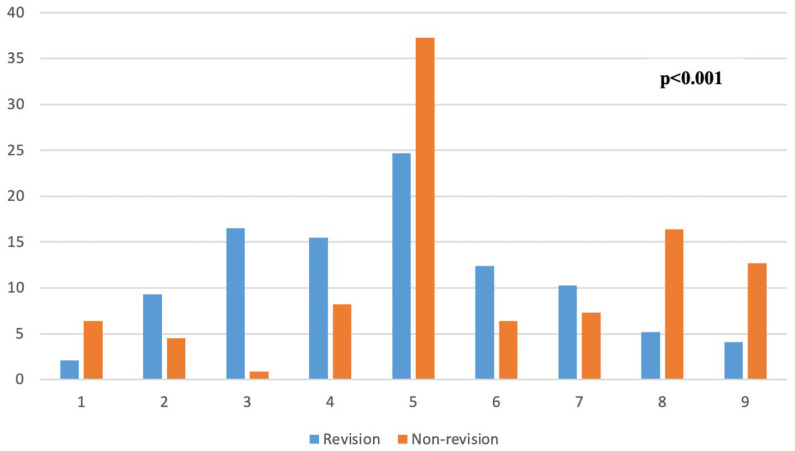
Comparison of screw positions according to Cleveland–Bosworth quadrants between two groups.

The most common screw position in both groups was 5 (37.3% of non-revision and 24.7% of revision patients), the center-center position according to Cleveland and Bosworth’s quadrants. A comparison of the screw positions between the two groups is given in Figure 2.

Cut-out was identified as the most common revision cause (*n* = 52, 53.6%), followed by loss of reduction (*n* = 15, 15.5%), implant failure (*n* = 14, 14.4%), nonunion (*n* = 6, 6.2%), infection (*n* = 4, 4.1%), cut-through (*n* = 3, 3.1%), and avascular necrosis of the femoral head (*n* = 3, 3.1%).

Total hip arthroplasty (*n* = 55, 56.7%) was the most common revision surgery, followed by hip hemiarthroplasty (*n* = 28, 28.9%), antibiotic hip spacer (*n* = 7, 7.2%), long pfna (*n* = 3, 3.2%), DHS (*n* = 1, 1%), pfna (*n* = 1, 1%), debridement alone (*n* = 1, 1%), and Girdlestone procedure (*n* = 1, 1%).

Median tip-apex distance was 25 mm (IQR, 19–31 mm) in the revision group and 22 mm (IQR, 17–29 mm) in the non-revision group, but this difference was not statistically significant (*p* = 0.168). Median calcar-referenced tip-apex distance was measured as 32 mm (IQR, 25–37 mm) in the revision group and 26 mm (IQR, 23–31 mm) in the non-revision group, and this difference was statistically significant (*p* < 0.001).

In the revision group, 31 patients had positive, 31 neutral, and 35 negative medial cortical support. In the non-revision group, 64 patients had positive, 34 neutral, and 12 negative medial cortical support, and this difference was statistically significant (*p* < 0.001).

Infection was seen in 15 patients (15.5%) in the revision group and in 1 patient (0.9%) in the non-revision group, and this difference was statistically significant (*p* < 0.001). Detailed information and comparison of patients’ demographic data, clinical data, and radiologic measurements are given in Table 1.

When bivariate analysis was performed to identify risk factors for revision, it was observed that female gender (*p* = 0.004), presence of posteromedial comminution (*p* < 0.001), operation under spinal anesthesia (*p* = 0.023), surgery in supine position (*p* < 0.001), using closed reduction techniques (*p* < 0.001), presence of infection (*p* = 0.004), and higher Charlson comorbidity index values (*p* < 0.001) increased the risk of revision. Additionally, positive and neutral medial cortex support (*p* < 0.001) decreased the risk of revision. Multivariate analysis was also applied to the parameters found to be significant in bivariate analysis. As a result of this analysis, surgery in supine position (*p* < 0.001), using closed reduction techniques (*p* < 0.001), and higher Charlson comorbidity index values (*p* < 0.001) remained significant while female gender, presence of posteromedial comminution, and operation under spinal anesthesia lost significance. Although the presence of infection was found to increase the risk of revision 14.48-fold, the wide confidence interval indicates a high degree of uncertainty regarding this association. Detailed results of bivariate and multivariate analysis are given in Table 2.

## 4. Discussion

This study evaluated the factors affecting the progression to revision in patients who underwent surgical treatment for intertrochanteric femur fractures. The findings show that fracture reduction is better in surgeries performed in the lateral decubitus position; however, revision rates are higher in this position compared to the supine position. This may be related to variables such as patient selection, fracture type, and surgical technique. In addition, the significantly lower revision rate in patients who underwent open reduction emphasizes the determinant role of reduction quality on surgical success. The high Charlson comorbidity index has emerged as an important factor that increases the risk of revision due to the difficulty of early mobilization of elderly patients. The presence of posteromedial comminution and negative medial cortical support variance have also been found to be significantly associated with higher revision rates. Classical tip-apex distance measurements have been found to be insufficient in predicting cut-out development alone.

In recent years, the frequency of using the lateral decubitus position in intertrochanteric femur fractures has increased in the literature [10]. In line with this trend, most of the surgeries in our hospital were performed in the lateral decubitus position. It has been reported in the literature that reduction is easier in the lateral decubitus position than in the supine position in unstable trochanteric fractures [11,12]. In the bivariate and multivariate analyses performed in this study, the revision rate was found to be significantly lower in surgeries performed in the lateral decubitus position than in the supine position. This may be due to the better fracture reduction in the lateral position. Similar studies in the literature also report that choosing the lateral decubitus position provides better outcomes [13].

In this study, the revision rate was found to be significantly higher in the closed reduction group than in the open reduction group. This may be due to the fact that a traction table was not used in any of the cases and that closed reduction did not provide optimal fracture reduction. With open reduction, a better quality reduction was achieved because direct access was provided to the fractured fragments. There is no consensus on this issue in the literature. It has been previously reported that closed reduction is superior [14], open reduction is superior [15], and union is dependent on the fracture type, regardless of the reduction type [16].

The mean Charlson comorbidity index was found to be significantly higher in the revision group. These higher values may reflect existing comorbidities and thus reduced mobility potential in patients. This situation may have caused a delay in early postoperative mobilization of patients and the development of fixation loss. A recent study in the literature reported that postoperative mortality was lower in patients who were mobilized early after hip fracture [17]. Similarly, a study with 1514 hip fractures showed that postoperative mobilization reduced mortality; however, no significant difference was found between full and partial weight bearing [18].

Many previously published studies have found the tip-apex distance to be a significant risk factor for cut-out, and the threshold for the tip-apex distance is generally found to be 25 mm [19,20,21]. A previous study stated that a calcar-referenced tip-apex distance of >31.75 mm increased the risk of cut-out. In the same study, it was shown that a classical tip-apex distance > 29.45 mm also increased the incidence of cut-out [22]. In this study, bivariate and multivariate analyses revealed that the classical and calcar-referenced tip-apex distances were not risk factors for PFN revision. This may be because the mean classical and calcar-referenced tip-apex distances of patients in both revision and non-revision groups were already less than 25 mm. Furthermore, the number of patients included may not have been sufficient to determine whether the tip-apex distance was a risk factor for implant failure.

The study examined the femoral medial cortical support variance and found that the positive variance was significantly higher in the non-revision group. This is consistent with the findings in the literature that positive medial cortical variance reduces revision rates and improves early functional outcomes [23]. Another study using finite element analysis suggested that negative medial cortical variance causes greater stress on the implant and may lead to earlier implant failure [24].

More surgical site infections were observed in the revision group, which may be explained by factors such as the need for a second surgery, more soft tissue dissection, longer operation time and dead space formation. Additionally, the higher prevalence of malnutrition and comorbidities in the elderly population with hip fractures may increase susceptibility to infection. There are many studies in the literature that are consistent with these findings [25,26,27,28].

In this study, posteromedial comminution was observed significantly more frequently in the revision group. This finding is consistent with the data in the literature indicating that the integrity of the posteromedial cortex plays a critical role in the stability of intertrochanteric fractures [23,29]. Posteromedial fragmentation, in particular, may lead to weakening of the medial column responsible for load transfer, which may negatively affect implant stability and increase the risk of complications such as nonunion or cut-out. Many studies have reported that in cases where the posteromedial cortex loses its integrity, mechanical stress is more concentrated on the implant, and this situation significantly increases the risk of surgical failure. At the same time, anatomic reduction becomes difficult in such fractures, and more aggressive surgical techniques are required. Especially in cases where lateral wall fracture and posteromedial comminution are present together, open reduction and additional fixation methods such as medial support plates are recommended [30,31]. In this study, posteromedial comminution was one of the most important anatomical factors associated with the need for revision, supporting previous literature.

When the effect of nail length and diameter on revision was evaluated, no significant difference was found between the groups. In the previous literature it was also reported that there was no significant difference in terms of complication and revision rates between modern designed short and long nails in the treatment of unstable intertrochanteric fractures [32,33].

When the diameter of the nails was compared in individuals with a wide femoral canal diameter in the geriatric patient group, no significant difference was found in terms of revision rates, fracture union, and complications, similar to the literature [34].

This study has some limitations. First, patient data were evaluated retrospectively, and some clinical or radiological parameters may have been missing. Second, the study is single-center and there may be technical differences between surgeons, and lack of standardization of preferences regarding patient positioning, reduction type, and implant type may have affected the results and caused bias. In addition, some technical details such as implant type and nail angle were not analyzed homogeneously. Although the reasons for revision were recorded in detail, long-term follow-up data such as biomechanical and functional outcomes are limited. Another limitation was that some 95% confidence intervals were wide due to the insufficient sample size. Therefore, the findings need to be supported by multicenter and prospective studies with a larger study sample.

## 5. Conclusions

Careful evaluation of fracture morphology, ensuring optimal reduction, and considering accompanying comorbidities of the patients in surgical planning of unstable trochanteric fractures stand out as key elements in increasing surgical success. When a traction table is not available, it is more beneficial to use a lateral decubitus position and open reduction to treat an unstable intertrochanteric fracture.

## Figures and Tables

**Table 1:** Demographic data, clinical data, and radiologic measurements of patients.

	Total (*n* = 207)	Revision (*n* = 97)	Non-Revision (*n* = 110)	*p*
Age, years, median (IQR)	81 (74–87)	82 (75.5–87)	78.5 (71–87)	0.095 ^¥^
Gender, *n* (%)				**0.004** ^a^
Female	137 (66.2)	74 (76.3)	63 (57.3)	
Male	70 (33.8)	23 (23.7)	47 (42.7)	
Fracture side, *n* (%)				0.738 ^a^
Left	105 (50.7)	48 (49.5)	57 (51.8)	
Right	102 (49.3)	49 (50.5)	53 (48.2)	
Dorr classification, *n* (%)				**<0.001** ^a^
Type A	23 (11.1)	23 (23.7)	0 (0)	
Type B	74 (35.8)	24 (24.7)	50 (45.5)	
Type C	110 (53.1)	50 (51.6)	60 (54.5)	
Posteromedial comminution, *n* (%)	85 (41.1)	53 (54.6)	32 (29.1)	**<0.001** ^a^
Anesthesia type, *n* (%)				**0.021** ^a^
Spinal	165 (79.7)	84 (86.6)	81 (73.6)	
General	42 (20.3)	13 (13.4)	29 (26.4)	
Patient’s position, *n* (%)				**<0.001** ^a^
Lateral decubitus	130 (62.8)	41 (42.3)	89 (80.9)	
Supine	77 (37.2)	56 (57.7)	21 (19.1)	
Reduction, *n* (%)				**<0.001** ^a^
Closed	170 (82.1)	65 (67)	105 (95.5)	
Open	37 (17.9)	32 (33)	5 (4.5)	
Infection, *n* (%)	16 (7.7)	15 (15.5)	1 (0.9)	**<0.001** ^a^
Medial cortical support variance, *n* (%)				**<0.001** ^a^
Neutral	65 (31.4)	31 (32.0)	34 (30.9)	
Positive	95 (45.9)	31 (32.0)	64 (58.2)	
Negative	47 (22.7)	35 (36.0)	12 (10.9)	
Trochanter minor fracture size, cm, median (IQR)	4 (3–5.4)	3.85 (2.9–5.1)	4 (3.1–5.5)	0.486 ^¥^
Calcar-referenced tip-apex distance, mm, median (IQR)	28 (23–35)	32 (25–37)	26 (23–31)	**<0.001** ^¥^
Classical tip-apex distance, mm, median (IQR)	24 (18–30)	25 (19–31)	22 (17–29)	0.168 ^¥^
Lateral cortical thickness, mm, median (IQR)	22 (14–29)	21 (13.8–29)	22.5 (14.8–29)	0.746 ^¥^
Charlson comorbidity index, mean ± SD	5.7 ± 1.7	6.3 ± 1.5	5.2 ± 1.7	**<0.001** ^b^

^a^ chi-square test, ^¥^ Mann–Whitney U test, ^b^ independent samples *t*-test, IQR: interquartile range, SD: standard deviation, cm: centimeter, mm: millimeter, bold indicates statistically significant results.

**Table 2:** Bivariate and multivariate analyses of the investigated parameters.

	Bivariate Analysis OR (95% CI)	*p*	Multivariate Analysis OR (95% CI)	*p*
Gender		**0.004**		0.066
Male	Ref		Ref	
Female	2.40 (1.31–4.38)		0.36 (0.12–1.06)	
Presence of posteromedial comminution		**<0.001**		0.666
No	Ref		Ref	
Yes	2.93 (1.66–5.21)		1.27 (0.42–3.85)	
Anesthesia type		**0.023**		0.112
General	Ref		Ref	
Spinal	2.31 (1.12–4.76)		3.16 (0.76–13.08)	
Patient’s position		**<0.001**		**<0.001**
Lateral decubitis	Ref		Ref	
Supine	5.78 (3.10–10.79)		9.13 (3.73–22.32)	
Reduction		**<0.001**		**<0.001**
Open	Ref		Ref	
Closed	10.33 (3.83–27.87)		24.98 (5.48–113.76)	
Surgical site infection		**0.004**		**0.029**
No	Ref		Ref	
Yes	19.93 (2.58–154.02)		14.48 (1.29–126.35)	
Medial cortical support		**<0.001**		0.630
Negative	Ref		Ref	
Neutral	0.16 (0.76–0.36)		0.32 (0.10–1.02)	
Positive	0.31 (0.13–0.70)		0.74 (0.22–2.43)	
Calcar-referenced tip-apex distance	1.01 (0.98–1.05)	0.292	1.08 (0.98–1.13)	0.225
Charlson comorbidity index	1.52 (1.24–1.86)	**<0.001**	2.19 (1.60–3.00)	**<0.001**

OR: odds ratio, CI: confidence interval, bold indicates statistically significant results.

## Data Availability

The original contributions presented in this study are included in the article. Further inquiries can be directed to the corresponding author.

## Abbreviations

The following abbreviations are used in this manuscript:

PFN	Proximal femoral nail
SHS	Sliding hip screw
TAD	Tip-apex distance
Cal TAD	Calcar-referenced tip-apex distance
IQR	Interquartile range
SD	Standard deviation
CI	Confidence interval
OR	Odds ratio
